# Autoradiography of ^3^H-pirenzepine and ^3^H-AFDX-384 in Mouse Brain Regions: Possible Insights into M_1_, M_2_, and M_4_ Muscarinic Receptors Distribution

**DOI:** 10.3389/fphar.2018.00124

**Published:** 2018-02-20

**Authors:** Paulina Valuskova, Vladimir Farar, Sandor Forczek, Iva Krizova, Jaromir Myslivecek

**Affiliations:** ^1^First Faculty of Medicine, Institute of Physiology, Charles University, Prague, Czechia; ^2^Institute of Experimental Botany, Academy of Sciences of the Czech Republic, Prague, Czechia

**Keywords:** M_1_ muscarinic receptor, M_2_ muscarinic receptor, M_4_ muscarinic receptor, ^3^H-pirenzepine, ^3^H-AFDX-384, ^3^H-QNB, autoradiography

## Abstract

Autoradiography helps to determine the distribution and density of muscarinic receptor (MR) binding sites in the brain. However, it relies on the selectivity of radioligands toward their target. ^3^H-Pirenzepine is commonly believed to label predominantly M_1_MR, ^3^H-AFDX-384 is considered as M_2_MR selective ligand. Here we performed series of autoradiographies with ^3^H-AFDX-384 (2 nM), and ^3^H-pirenzepine (5 nM) in WT, M_1_KO, M_2_KO, and M_4_KO mice to address the ligand selectivity. Labeling with ^3^H-pirenzepine using M_1_KO, M_2_KO, and M_4_KO brain sections showed the high selectivity toward M_1_MR. Selectivity of ^3^H-AFDX-384 toward M_2_MR varies among brain regions and depends on individual MR subtype proportion. All binding sites in the medulla oblongata and pons, correspond to M_2_MR. In caudate putamen, nucleus accumbens and olfactory tubercle, 77.7, 74.2, and 74.6% of ^3^H-AFDX-384 binding sites, respectively, are represented by M_4_MR and M_2_MR constitute only a minor portion. In cortex and hippocampus, ^3^H-AFDX-384 labels almost similar amounts of M_2_MR and M_4_MR alongside significant amounts of non-M_2_/non-M_4_MR. In cortex, the proportion of ^3^H-AFDX-384 binding sites attributable to M_2_MR can be increased by blocking M_4_MR with MT3 toxin without affecting non-M_4_MR. PD102807, which is considered as a highly selective M_4_MR antagonist failed to improve the discrimination of M_2_MR. Autoradiography with ^3^H-QNB showed genotype specific loss of binding sites. In conclusion: while ^3^H-pirenzepine showed the high selectivity toward M_1_MR, ^3^H-AFDX-384 binding sites represent different populations of MR subtypes in a brain-region-specific manner. This finding has to be taken into account when interpreting the binding data.

## Introduction

Muscarinic receptors (MR) are typical members of G protein coupled receptors family (Kruse et al., 2013) and can be divided into 5 subtypes (M_1_–M_5_) (Eglen, 2012), which activate different G proteins (G_q_, G_i_). Odd-numbered subtypes activate G_q_, even-numbered activate G_i_ protein (Eglen, 2012; Kow and Nathanson, 2012; Reiner and Nathanson, 2012).

Respective MR subtypes have been assigned to different functions in CNS (Wess et al., 2007; Thomsen et al., 2017). Odd-numbered receptors are considered to be localized primarily post-synaptically, however, both M_2_MR and M_4_MR are localized both pre- and post-synaptically. As cholinergic autoreceptors, M_2_ and M_4_ provide feedback control of acetylcholine release (Zhang et al., 2002; Shin et al., 2015).

One of the means to determine MR density in different CNS areas is autoradiography. *In vitro* autoradiography has high sensitivity allowing to explore brain regions even with few MR. The use of very thin tissue sections *in vitro* autoradiography provides several advantages over the large tissue blocks used in binding studies in homogenates/membrane fractions. Brain sectioning allows analyzing MR density in virtually all brain areas of a single animal greatly reducing the number of experimental animals. Moreover, sectioning of a single brain generates sufficient number of tissue sections to explore the binding of multiple radioligands in a particular brain area of the same animal. This further reduces the number of experimental animals and allows comparing the effect of treatment on multiple targets (receptors, transporters) in a single animal (Farar and Myslivecek, 2016).

An important issue is the selectivity of radioligand used in experiments. A general problem in identification of MR subtypes present in specific regions of the central nervous system is the lack of highly subtype-selective muscarinic antagonists. The MR subtypes affinities for pirenzepine and AFDX-384, the most commonly used ones for discrimination of M_1_ and M_2_MR, respectively, are shown in Table 1. It can be deduced from this table that both pirenzepine and AFDX-384 have not only high affinity for M_1_, and M_2_ MRs, respectively, but also for M_4_ MR subtype. In radioligand binding studies, it is therefore necessary to use a combination of various antagonists. However, for autoradiography detection this approach is not suitable because of evaluation limitations of such changed “binding.” Thus, the present protocols for M_1_ and M_2_ MR subtypes identification should be considered as method for detection of M_1_ (or M_2_) and also yet unidentified portion of M_4_ MRs. Unfortunately, only few papers report these binding sites as M_2_/M_4_ MRs (e.g., Zavitsanou et al., 2003; Wang et al., 2014).

**Table 1:** Muscarinic antagonist affinity constants (log affinity or pKi values) for mammalian muscarinic receptor subtypes.

	**Receptor subtype**				
**Antagonist**	**M_1_**	**M_2_**	**M_3_**	**M_4_**	**M_5_**
Pirenzepine	7.8–8.5	6.3–6.7	6.7–7.1	7.1–8.1	6.2–7.1
AF-DX 384	7.3–7.5	8.2–9.0	7.2–7.8	8.0-8.7	6.3
PD 102807	5.3–5.5	5.7–5.9	6.2–6.7	7.3–7.4	5.2–5.5
MT 7 toxin	9.8	<6	<6	<6	<6
MT 3 toxin	7.1	<6	<6	8.7	<6

*Data as referenced by Myslivecek et al. (2008)*.

The lack of MR subtype-selective ligands constitutes a significant obstacle in anatomical localization studies of MR, which are fundamental to our understanding of MR neuronal circuits. Three key strategies were developed to address regional distribution and relative abundance of particular MR subtype in the central nervous system.

First, *in situ* hybridization studies have identified neurons that synthetize MR (Buckley et al., 1988; Vilaró et al., 1990; Weiner et al., 1990). These and several other studies have shown that all five MR subtypes are expressed in the brain and mRNA for an individual MR subtype is distributed in a brain-region specific manner. However, while *in situ* hybridization studies provide valuable information about the sites of MR expression they do not address the real distribution of final proteins.

Second, MR subtype selective antibodies have been developed to map and quantify the distribution of individual MR by means of immunocytochemistry and immunoprecipitation (Levey et al., 1991; Yasuda et al., 1993). A body of reports have provided information about the distribution and quantities of individual MR. However, the reliability and selectivity of commonly used antibodies against MR have been questioned by their testing in specimens devoidof particular MR subtypes. This knockout-proof specific labeling has shown that the vast majority of tested antisera have identical labeling patterns in wild-type and knockout mice tissues (Jositsch et al., 2009).

Finally, anatomical localization and relative quantification of MR can be done by means *of in vitro* radioligand binding studies. This includes direct radioligand binding studies in tissue homogenates or plasma membrane preparation and indirect *in vitro* autoradiographic assays. *In vitro* autoradiography offers several advantages over direct binding studies. These includes tissue saving, high precision and reproducibility of results, high sensitivity and high degree of anatomical resolution. Moreover several ligands can be applied on consecutive sections derived from the same animal, further reducing numbers of experimental animals (Farar and Myslivecek, 2016).

Historically, tritiated pirenzepine was used as ligand that binds to MR with distinct binding in specific brain areas (Yamamura et al., 1983). Further, distinct distribution was found in the central nervous system. ^3^H-pirenzepine labels regions of the cerebral cortex, hippocampus, striatum and dorsal horn of the spinal cord, while sites in the cerebellum, nucleus tractus solitarius, facial nucleus and ventral horn of the spinal cord are labeled with ^3^H-QNB (non-specific muscarinic ligand) and not by ^3^H-pirenzepine (Wamsley et al., 1984). These observations indicated binding to different subtypes of MR. This was further expanded to definition of binding sites as M_1_ MR (Villiger and Faull, 1985). In the middle of eighties pirenzepine binding sites were considered as M_1_ MR (Buckley and Burnstock, 1986; Cortes and Palacios, 1986). On the other hand, ^3^H-AFDX-384 was considered from the beginning as a M_2_ MR specific ligand (Aubert et al., 1992) and some authors were aware of limited selectivity (e.g., Mulugeta et al., 2003). In many cases^3^H-AFDX-384 and ^3^H-pirenzepine are still considered as selective ligands (Tien et al., 2004; Wolff et al., 2008).

Here we performed series of *in vitro* autoradiographies with ^3^H-AFDX-384 (2 nM, concentration below K_D_ and the most commonly used one), and ^3^H-pirenzepine (5 nM, concentration below K_D_ and the most used one). We took advantage of knockout mice models in the standard autoradiography procedures to compare binding in WT, M_1_ KO, M_2_ KO and M_4_ KO mice. With this approach we addressed the selectivity of *in vitro* ^3^H-AFDX-384 autoradiography and provide a guide on the interpretation of results.

Moreover, we tried to block M_4_MR using three concentration of MT3 toxin isolated from Dendroaspis angusticeps venom (1, 10, and 100 nmol/l). Another method to disable the binding to M_4_MR was co-incubation with specific M_4_MR antagonist PD102807 (10, 100, and 1 μmol/l). With an aim to block M_1_MR we used MT7 toxin isolated from Dendroaspis angusticeps venom (1, 10, or 100 nmol/l) or pirenzepine (10 and 100 nmol/l).

In another of our experiment, using radiolabeled non-selective anatagonist, which binds all five MR subtypes (^3^H-QNB), we determined the contribution of M_1_, M_2_, and M_4_ MRs to the total expression of MRs in the mouse brain.

We can conclude that ^3^H-pirenzepine showed high selectivity toward M_1_MR. In contrast, ^3^H-AFDX-384 binding sites represent different populations of MR subtypes in a brain-region-specific manner. This finding has to be taken into account when interpreting the binding data not only in the autoradiographies but also when these antagonists (pirenzepine, AFDX-384, PD102807 or MT3 toxin) are used as a mean to detect M_1_MR, M_2_MR, or M_4_MR effects in functional studies. Experiments with ^3^H-QNB binding decrease in M_1_, ${\text{M}}_{2^{\cdot \cdot }}$, and M_4_ KO animals showed the highest proportion (usually above 50%) of M_1_MR in virtually all studied brain areas. M_2_MR take up to 20% in cortical areas and 34% in thalamus. M_4_MR were abundant (40% approximately) in thalamus, striatum and ventral striatum (NAc and OT), about 20% of M_4_MR can be found in cortical structures.

## Methods

### Drugs

Atropine sulfate and pirenzepine dihydrochloride were purchased from Sigma-Aldrich (Sigma-Aldrich Co, St. Louis, MO, USA). PD102807 was purchased from Tocris Bioscience (Tocris Bioscience, Bristol, United Kingdom). MT3 toxin and MT7 toxin were purchased from Peptide Institute (Peptide Institute, Inc., Osaka, Japan). Pirenzepine [N-methyl-^3^H] (83.4 Ci/mmol), and AFDX-384 [2,3-dipropylamino-3H] (100.0 Ci/mmol) were from American Radiolabeled Chemicals (ARC, Inc.), Qinuclidinyl benzilate L-[benzilic-4,4′-^3^H] (50.5 Ci/mmol) was purchased from Perkin Elmer (Perkin Elmer Inc., USA).

### Animals

Mice were treated in accordance with the legislature of the Czech Republic and EU legislature [European Convention for the Protection of Vertebrate Animals used for Experimental and other Scientific Purposes (Council of Europe No 123, Strasbourg 1985)], and the experimental protocol was approved by the Committee for the Protection of Experimental Animals of the 1st Medical Faculty, Charles University, Prague under N° MSMT-6316/2014-39.

Mice were maintained (3 per cage) under controlled environmental conditions (12 h/12 h light/dark cycle, 22±1°C, light on at 06:00 a.m.). Food and water were available *ad libitum*. Knockout mice and their WT counterparts of both genders (weighting 20–25 g, 11–13 weeks old), were used in the study. We studied fully backcrossed (at least 10 generations) muscarinic KO and WT littermates.

#### M_1_ KO mice

Mice lacking M_1_ MR subtype were generated in the Wess laboratory (Bymaster et al., 2003) and then bred in our animal facility (Prague, Czech Republic). Their genetic background was C57Bl6/NTac. WT and KO genotypes were confirmed using polymerase chain reaction (PCR) analysis as previously described (Cea-del Rio et al., 2010).

#### M_2_ KO mice

Mice lacking M_2_ MR subtype was generated in the Wess laboratory (Gomeza et al., 1999a) and then bred in our animal facility (Prague, Czech Republic). The genetic background was maintained on C57Bl6/NTac mouse line. WT and KO genotypes were confirmed using PCR analysis as previously described (Cea-del Rio et al., 2010).

#### M_4_ KO mice

Mice lacking M_4_ MR subtype were generated in Wess laboratory (Gomeza et al., 1999b) and then bred in our animal facility (Prague, Czech Republic). Their genetic background was C57Bl6/NTac. WT and KO genotypes were confirmed using PCR analysis as previously described (Cea-del Rio et al., 2010).

### Receptor autoradiography

#### Tissue preparation

For receptor determination, autoradiography was performed in several brain areas [motor cortex (MOCx), somatosensory cortex (SSCx), visual cortex (VisCx), striatum (Caudatum-Putamen, CPu), nucleus accumbens (NAc), thalamus (TH), hippocampus (Hipp) and its specific areas CA1, CA3 and dentate gyrus (DG), olfactory tubercle (OT), pons (Pons), and medulla oblongata (MY)] on sagittal brain sections of M_1_KO, M_2_KO, M_4_KO mice and their WT littermates. Brains were rapidly removed (4–6 brains per group), frozen on dry ice, and then stored at −80°C until cryostat sectioning. Sixteen-micrometer thick sagittal sections were cut on a cryostat at −20°C and thaw-mounted on Superfrost® Plus glass slides (Carl Roth GmbH & Co. KG, Karlsruhe, Germany) and stored in storage boxes at −80°C until use. For autoradiography experiments brain sections were allowed to thaw and dry for 30 min at 22°C.

#### Autoradiography of muscarinic receptors on M_1_KO, M_2_KO, M_4_KO, and WT mice (^3^H-pirenzepine, ^3^H-AFDX-384, and ^3^H-QNB radioligand binding)

Brain sections were allowed to thaw and dry for 30 min at 22°C and density of receptors was determined as previously described (Farar et al., 2012; Farar and Myslivecek, 2016). Dry M_1_KO M_2_KO, M_4_KO, and WT sagittal brain sections were pre-incubated for 30 min in 50 mM potassium phosphate buffer (pH 7.4) at room temperature (RT). Following pre-incubation, sections were transferred into fresh 50 mM potassium phosphate buffer containing 2 nM ^3^H-AFDX-384, or 5 nM ^3^H-pirenzepine, or 2 nM ^3^H-QNB, and incubated for 60 min at RT. Non-specific binding was assessed on adjacent sections in the presence of 10 μM atropine sulfate. After incubation, sections were washed two times for 5 min each in ice-cold buffer and dipped for 2 s in ice-cold water to remove buffer salts. Wet sections were immediately dried by gentle stream of cold air. Dry sections were opposed to tritium sensitive Fuji BAS imaging plates (GE Healthcare Europe GmbH, Freiburg, Germany) in Kodak Biomax autoradiographic cassettes (Carestream Health, Inc., Rochester, NY).

#### Autoradiography of M_2_ muscarinic receptors labeled with ^3^H-AFDX-384 and simultaneous blocking of M_4_ receptors with PD102807

The procedure was similar as described above. However, the sections were transferred into buffer containing 2 nM ^3^H-AFDX-384 together with 10; 100 or 1,000 nM PD102807 or without PD102807 but with addition of DMSO which serves as dissolving agent for PD102807 and incubated for 60 min at RT. The final concentration of DMSO in incubation buffer must be less than 1%, in our case it was 0.1%. The non-specific binding was assessed as described above. This specific experiment was conducted on M_2_ KO and M_4_ KO brain sections only. After incubation, sections were treated as stated above.

#### Autoradiography of M_2_ muscarinic receptors labeled with ^3^H-AFDX-384 and simultaneous blocking of M_4_ receptors with MT3 toxin isolated from dendroaspis angusticeps venom

The procedure was similar as described above. However, the sections were transferred into buffer containing 2 nM ^3^H-AFDX-384 together with 1; 10 or 100 nM MT3 toxin or without MT3 toxin and incubated for 60 min at RT. Non-specific binding was assessed as described above. After incubation, sections were treated as described above.

#### Autoradiography of M_2_ muscarinic receptors labeled with ^3^H-AFDX-384 and simultaneous blocking of M_1_ receptors with MT7 toxin isolated from dendroaspis angusticeps venom or with pirenzepine

The procedure was similar as described above. However, the sections were transferred into buffer containing 2 nM ^3^H-AFDX-384 together with 10 or 100 nM pirenzepine or without pirenzepine (serves as control) and incubated for 60 min at RT. Blocking of M_1_ MR with MT7 toxin was conducted as following: the brain sections were at first pre-incubated with different concentrations of MT7 toxin (1; 10 or 100 nM) for 45 min. After pre-incubation with MT7, ^3^H-AFDX-384 with final concentration 2 nM was added into the buffer containing MT7 toxin and incubated for another 45 min. Non-specific binding as mentioned earlier. After incubation, sections were processed as described above.

#### Quantification of receptor density

To assure linearity of the signal, autoradiographic standards (American Radiolabeled Chemicals, Inc., St. Louis, MO, USA) were exposed along with the samples to the screens. Imaging plates were processed in Fuji Bioimaging Analyzer BAS-5000 (FUJIFILM corporation, Tokio, Japan) and digitized images analyzed with MCID analysis software (InterFocus GmbH, Mering, Germany). Measurements were taken and averaged from three sections for each animal and brain region.

### Statistical analysis

Statistical significance between groups was determined using 1-way ANOVA with Sidak *post-hoc* analysis. Student's *t*-test was used for comparison between two groups (typically WT vs. KO only).

## Results

### The selectivity of ^3^H-pirenzepine toward M_1_MR

**Figure 1:**
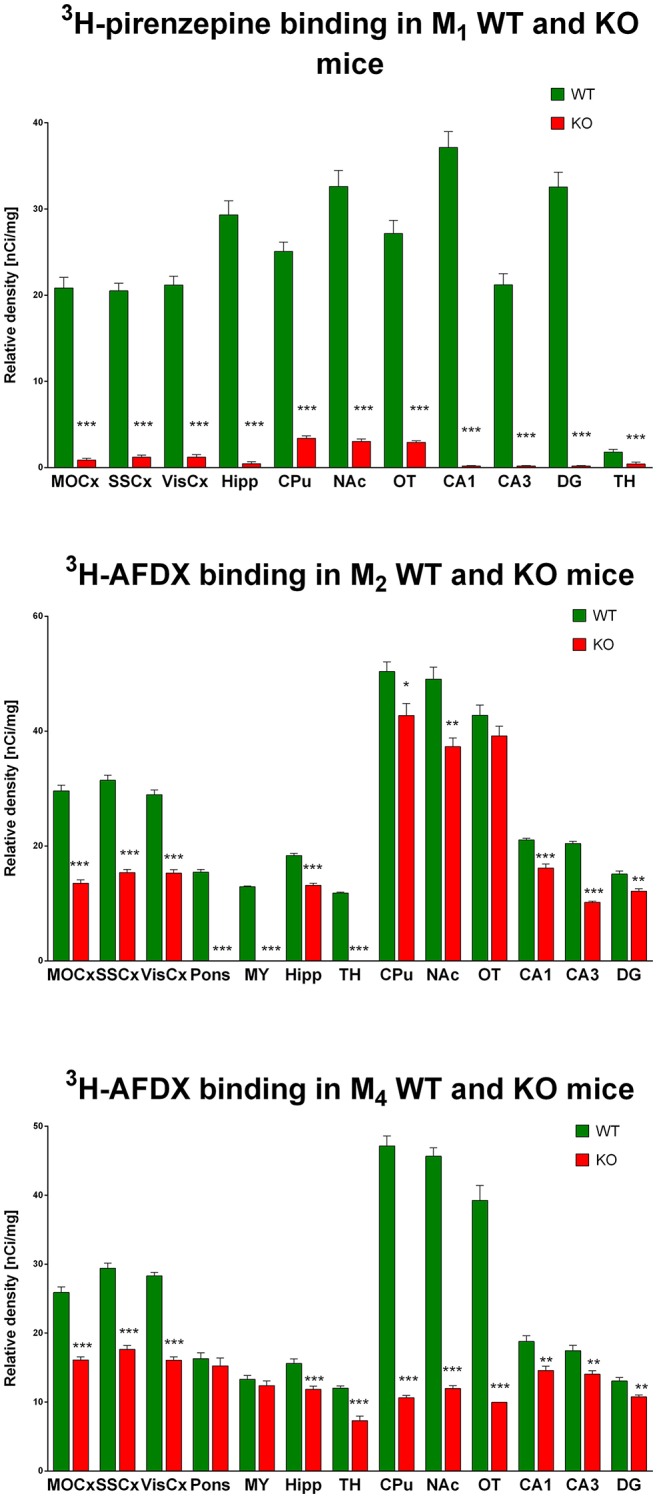
Changes in ^3^H-pirenzepine specific binding in M_1_KO mice (**top**) and ^3^H-AFDX binding in M_2_KO (**middle**) and M_4_KO mice (**bottom**) when compared to WT animals. Non-specific binding was determined in the presence of 10 μM atropine sulfate. Ordinate: brain areas [motor cortex (MOCx), somatosensory cortex (SSCx), visual cortex (VisCx), striatum (Caudatum-Putamen, CPu), nucleus accumbens (NAc), thalamus (TH), hippocampus (Hipp) and its specific areas CA1, CA3, and dentate gyrus (DG), olfactory tubercle (OT)], abscissa: relative density [nCi/mg]. WT, wild type; KO, knockout mice. ^*^p < 0.05, ^**^p < 0.01, ^***^p < 0.001.

Autoradiography with ^3^H-pirenzepine showed high selectivity of this ligand toward M_1_MR (see Figure 1). In many brain areas, except in CPu and OT the binding in M_1_ KO animals decreased by 90–99% (see Table 2). Especially, in hippocampal areas (dorsal hippocampus, CA1, CA3 area, and DG) ^3^H-pirenzepine binding sites were almost completely abolished. Please note very low binding in TH. The selectivity of pirenzepine was confirmed in M_2_ KO animals (see Table 3) where no significant difference was shown in M_2_ KO animals when compared to WT animals. Similar results were obtained when using M_4_ KO animals (see Table 4). However, in some brain areas, we found small, but significant decrease in ^3^H-pirenzepine binding in M_4_ KO animals (MOCx, SSCx, VisCx, CPu; 7, 13, 9, and 11%, respectively).

**Table 2:** Percentage difference in ^3^H-pirenzepine binding in M_1_KO, and in ^3^H-AFDX-384 to M_2_KO, and M_4_KO animals to WT animals.

**M**_**1**_**KO (**^**3**^**H-pirenzepine)**			**M**_**2**_**KO (**^**3**^**H-AFDX-384)**		**M**_**4**_**KO (**^**3**^**H-AFDX-384)**	
**Brain area**	**Difference from WT [%]**	**Significance (*p*)**	**Difference from WT[%]**	**Significance (*p*)**	**Difference from WT[%]**	**Significance (*p*)**
MOCx	−95.85	<0.0001	−54.38	<0.0001	−37.82	<0.0001
SSCx	−94.14	<0.0001	−51.08	<0.0001	−40.04	<0.0001
VisCx	−94.35	<0.0001	−47.19	<0.0001	−43.23	<0.0001
Hipp	−98.49	<0.0001	−28.32	<0.0001	−24.03	=0.00100
TH	−76.68	=0.0096	−100.00	<0.0001	−39.31	=0.0001
CPu	−86.49	<0.0001	−15.19	=0.0290	−77.46	<0.0001
NAc	−90.71	<0.0001	−23.99	=0.0036	−73.78	<0.0001
OT	−89.23	<0.0001	−8.48	NS	−74.62	<0.0001
CA1	−99.48	<0.0001	−23.19	=0.0007	−22.41	0.0022
CA3	−99.10	<0.0001	−50.10	<0.0001	−19.64	=0.0064
DG	−99.41	<0.0001	−19.86	=0.0028	−17.62	=0.0077

*Statistical significance determined using unpaired Student t-test. For abbreviations see text*.

**Table 3:** Binding and percentage difference in ^3^H-pirenzepine binding in M_2_KO animals and WT animals.

**Brain area**	**Relative density [nCi/mg]**		**Difference from WT (%)**	**Significance (*p*)**
	**M_2_WT (x ± SEM)**	**M_2_KO (x ± SEM)**		
MOCx	37.27 ± 1.11	37.50 ± 0.67	1.46	NS
SSCx	37.31 ± 1.15	36.20 ± 0.73	1.01	NS
VisCx	38.81 ± 1.54	39.41 ± 0.89	0.82	NS
Hipp	57.32 ± 1.66	58.46 ± 0.90	1.32	NS
CPu	47.87 ± 1.75	48.52 ± 0.91	2.01	NS
NAc	56.57 ± 2.30	54.51 ± 1.70	1.54	NS
OT	47.13 ± 2.87	50.25 ± 1.02	2.60	NS
CA1	65.45 ± 1.71	66.03 ± 0.84	1.26	NS
CA3	43.46 ± 0.95	43.66 ± 0.38	1.32	NS
DG	58.85 ± 2.00	58.95 ± 0.89	1.42	NS

*Statistical significance determined using unpaired Student t-test. For abbreviations see text*.

**Table 4:** Binding and percentage difference in ^3^H-pirenzepine binding in M_4_KO animals and WT animals.

**Brain area**	**Relative density [nCi/mg]**		**Difference from WT (%)**	**Significance**
	**M_4_WT (x ± SEM)**	**M_4_KO (x ± SEM)**		
MOCx	36.86 ± 0.78	34.39 ± 0.67	−6.71	=0.0369
SSCx	35.98 ± 1.13	31.26 ± 0.73	−13.11	=0.0057
VisCx	37.32 ± 1.16	34.02 ± 0.89	−8.83	=0.0473
Hipp	54.94 ± 1.45	54.99 ± 0.90	0.10	NS
CPu	47.85 ± 1.65	42.46 ± 0.91	−11.27	=0.0170
NAc	55.20 ± 1.65	52.81 ± 1.70	−4.33	NS
OT	46.58 ± 2.85	42.10 ± 1.02	−9.61	NS
CA1	62.96 ± 1.31	62.78 ± 0.84	−0.28	NS
CA3	44.48 ± 1.35	44.00 ± 0.38	−1.07	NS
DG	55.28 ± 1.73	55.97 ± 0.89	1.25	NS

*Statistical significance determined using unpaired Student t-test. For abbreviations see text*.

### The selectivity of ^3^H-AFDX-384 toward M_2_MR

In contrast to the above mentioned experiment, ^3^H-AFDX-384 did not show selectivity toward M_2_MR. If ^3^H-AFDX-384 was highly selective toward M_2_MR, the binding of ^3^H-AFDX-384 would be barely visible in M_2_ KO mice, as they do not express M_2_MR. As it can be deduced from Figure 1 and Table 2, the binding reduction in many brain areas varied between 20 and 50%, with exception of pons, medulla oblongata and TH, where uniform population of M_2_MR was found. Olfactory tubercle is the only area, in which the binding of ^3^H-AFDX-384 was completely preserved.

### The selectivity of ^3^H-AFDX-384 toward M_4_MR

**Figure 2:**
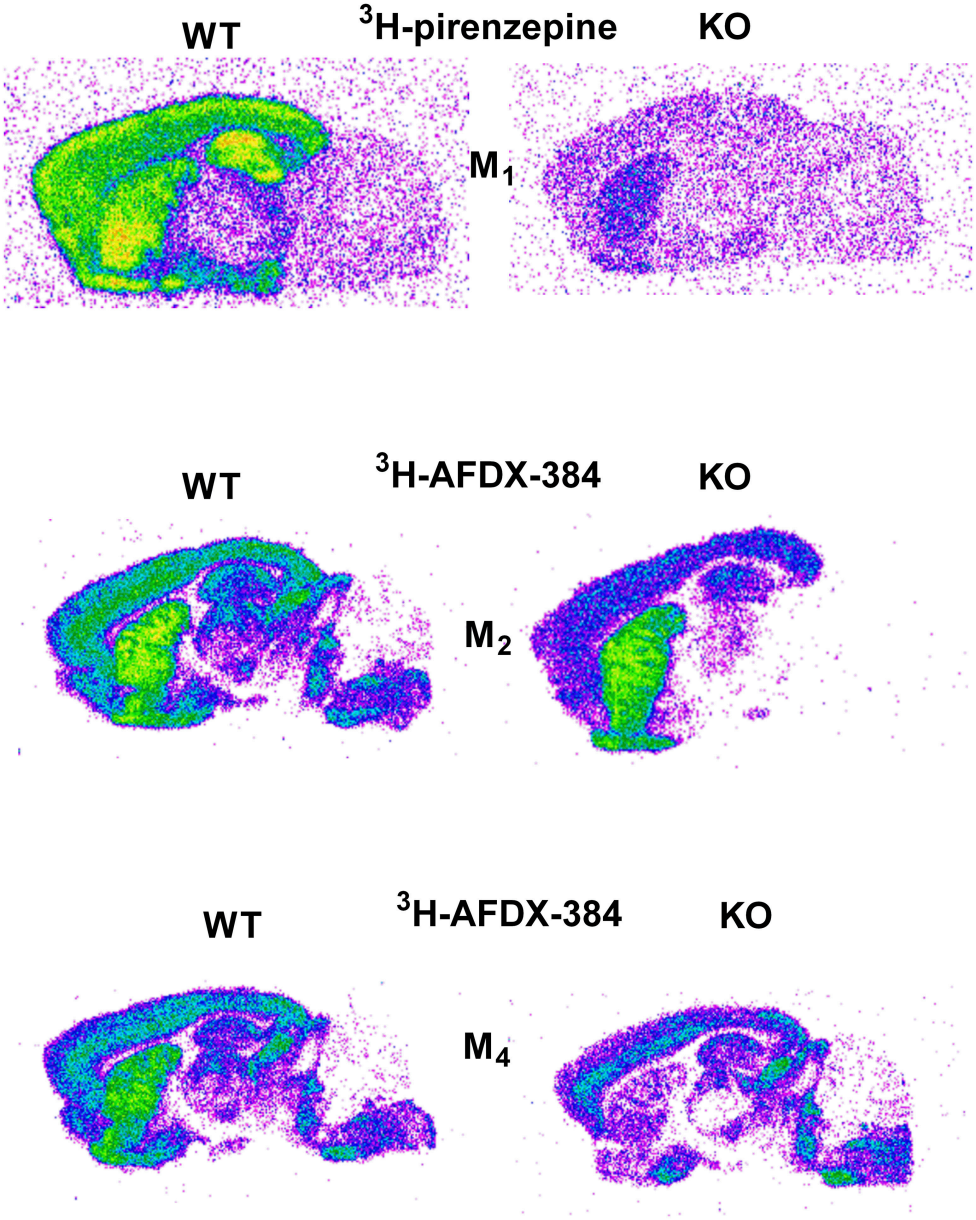
Representative autoradiograms in WT and KO mice: ^3^H-pirenzepine binding in M_1_ WT and KO mice (**top**), ^3^H-AFDX 384 binding in M_2_ WT and KO mice (**middle**), and ^3^H-AFDX 384 binding in M_4_ WT and KO mice (**bottom**).

Similar to the experiment in M_2_ KO mice, the binding in M_4_ KO mice showed only limited selectivity of ^3^H-AFDX-384 toward this MR subtype. However, ^3^H-AFDX-384 had similar selectivity to M_4_MR and M_2_MR (see Figure 1 and Table 2). A decrease in ^3^H-AFDX-384 binding depends on the brain area analyzed. This experiment also verified that pons and medulla oblongata express only M_2_MR which can be deduced from the unchanged binding in M_4_ KO mice. However, thalamus was not confirmed as an area with only M_2_MR. Representative autoradiograms for M_1_, M_2_ and M_4_ WT and KO mice are shown in Figure 2.

### Binding of ^3^H-AFDX-384 in the presence of M_4_MR antagonist PD102807

The effort to block M_4_MR using antagonist PD102807 was unsuccessful as demonstrated in M_2_ KO mice and M_4_ KO mice. When the slices were incubated with 1 μmol/l of PD102807, we have recorded 100% inhibition of residual binding in M_2_ KO mice (see Table 5). However, PD102807 also dose-dependently reduced ^3^H-AFDX-384 binding in M_4_ KO mice, suggesting limited selectivity toward M_4_MR.

**Table 5:** Binding and percentage difference in ^3^H-AFDX 384 binding with PD102807 in M_2_KO, and M_4_KO animals vs. control (no PD102807).

**Brain area**		**M_2_KO [nCi/mg] x ± SEM**	**M_2_KO control vs. M_2_KO PD**	***P*-value**	**M_4_KO [nCi/mg] x ± SEM**	**M_4_KO control vs. M_4_KO PD**	***P*-value**
MOCx	Control	13.76 ± 1.388			16.78 ± 1.060		
	10 nM PD	5.668 ± 0.053	−58.81	<0.001	12.67 ± 1.482	−24.49	=0.021
	100 nM PD	5.365 ± 0.051	−61.01	<0.001	12.35 ± 0.458	−26.40	= 0.029
	1000 nM PD	0 ± 0.000	−100.00	<0.001	8.157 ± 1.140	−51.39	<0.001
SSCx	Control	17.25 ± 1.977			18.42 ± 1.460		
	10 nM PD	6.654 ± 0.882	−61.43	<0.001	13.79 ± 0.923	−25.14	=0.017
	100 nM PD	5.325 ± 0.057	−69.13	<0.001	11.11 ± 1.216	−39.69	=0.002
	1000 nM PD	0 ± 0.000	−100.00	<0.001	8.563 ± 1.040	−53.51	<0.001
VisCx	Control	16.39 ± 2.554			16.66 ± 1.067		
	10 nM PD	6.858 ± 0.530	−58.16	<0.001	13.22 ± 1.444	−20.65	=0.091
	100 nM PD	5.769 ± 0.078	−64.80	<0.001	9.092 ± 1.588	−45.43	=0.003
	1000 nM PD	0 ± 0.000	−100.00	<0.001	6.69 ± 1.117	−59.84	<0.001
Hipp	Control	14.82 ± 1.028			12.76 ± 0.464		
	10 nM PD	7.857 ± 0.726	−46.98	<0.001	8.926 ± 0.976	−30.05	=0.005
	100 nM PD	5.972 ± 0.406	−59.70	<0.001	6.632 ± 0.670	−48.03	<0.001
	1000 nM PD	0 ± 0.000	−100.00	<0.001	6.233 ± 0.966	−51.15	<0.001
CPu	Control	48.23 ± 3.175			10.69 ± 1.012		
	10 nM PD	31.33 ± 0.238	−35.04	<0.001	6.896 ± 1.074	−35.49	=0.006
	100 nM PD	20.44 ± 1.202	−57.62	<0.001	6.123 ± 0.551	−42.72	=0.003
	1000 nM PD	0 ± 0.000	−100.00	<0.001	5.598 ± 0.238	−47.63	=0.002
NAc	Control	43.92 ± 4.107			11.52 ± 1.251		
	10 nM PD	31.42 ± 2.005	−28.46	<0.001	8.234 ± 1.101	−28.52	=0.029
	100 nM PD	18.1 ± 0.674	−58.79	<0.001	6.641 ± 0.634	−42.35	=0.006
	1000 nM PD	0.168 ± 0.058	−99.62	=0.002	6.478 ± 0.588	−43.77	=0.008
OT	Control	44.55 ± 4.201			7.048 ± 0.399		
	10 nM PD	21.73 ± 1.338	−51.22	<0.001	5.452 ± 0.212	−22.64	<0.001
	100 nM PD	8.894 ± 0.253	−80.04	<0.001	0.425 ± 0.145	−93.97	<0.001
	1000 nM PD	0 ± 0.000	−100.00	<0.001	0 ± 0.000	−100.00	<0.001

*Statistical significance determined using one-way ANOVA with post-hoc Sidak analysis. PD, PD102807. For other abbreviations see text*.

### Binding of ^3^H-AFDX-384 in the presence of M_4_MR specific toxin MT3

Similar to PD102807, MT3 toxin was unable to block M_4_MR. This can be seen in competitions binding experiment with increasing concentration of MT3 toxin (Table 6). MT3 toxin was unable to completely block residual ^3^H-AFDX-384 binding in M_2_ KO mice. Moreover, MT3 also dose-dependently reduced ^3^H-AFDX-384 binding in M_4_ KO mice.

**Table 6:** Binding and percentage difference in ^3^H-AFDX 384 binding with MT3 toxin in M_2_KO, and M_4_KO animals vs. control (no MT3 toxin).

**Brain area**		**M_2_KO [nCi/mg] x ± SEM**	**M_2_KO control vs. M_2_KO MT3**	***P*-value**	**M_4_KO [nCi/mg] x ± SEM**	**M_4_KO control vs. M_4_KO MT3**	***P*-value**
MOCx	Control	11.01 ± 0.234			15.61 ± 0.821		
	1 nM MT3	9.32 ± 0.422	−15.35	<0.001	14.81 ± 0.427	−5.12	NS
	10 nM MT3	4.468 ± 0.125	−59.42	<0.001	13.86 ± 1.874	−11.21	NS
	100 nM MT3	2.115 ± 0.081	−80.79	<0.001	10.57 ± 0.470	−32.29	=0.018
SSCx	Control	13.42 ± 0.468			15.15 ± 2.011		
	1 nM MT3	10.97 ± 0.870	−18.26	=0.009	15.95 ± 0.509	5.28	NS
	10 nM MT3	5.173 ± 0.479	−61.45	<0.001	13.17 ± 1.678	−13.07	NS
	100 nM MT3	2.393 ± 0.145	−82.17	<0.001	11.08 ± 0.514	−26.86	NS
VisCx	Control	13.59 ± 0.822			14.79 ± 2.159		
	1 nM MT3	10.52 ± 0.617	−22.59	=0.005	14.97 ± 1.241	1.22	NS
	10 nM MT3	4.949 ± 0.648	−63.58	<0.001	13.08 ± 2.405	−11.56	NS
	100 nM MT3	2.177 ± 0.361	−83.98	<0.001	11.69 ± 1.124	−20.96	NS
Hipp	Control	11.37 ± 0.445			13.37 ± 1.158		
	1 nM MT3	9.061 ± 0.217	−20.31	<0.001	12.29 ± 0.925	−8.08	NS
	10 nM MT3	5.851 ± 0.436	−48.54	<0.001	9.094 ± 0.503	−31.98	=0.006
	100 nM MT3	2.824 ± 0.227	−75.16	<0.001	7.16 ± 0.489	−46.45	<0.001
CPu	Control	31.59 ± 1.035			9.659 ± 0.128		
	1 nM MT3	26.94 ± 1.756	−14.72	=0.01	8.931 ± 0.192	−7.54	NS
	10 nM MT3	8.836 ± 0.641	−72.03	<0.001	7.81 ± 1.325	−19.14	NS
	100 nM MT3	3.03 ± 0.275	−90.41	<0.001	5.041 ± 0.129	−47.81	=0.001
NAc	Control	30.7 ± 0.446			12.19 ± 0.071		
	1 nM MT3	24.85 ± 1.185	−16.49	=0.001	10.42 ± 0.722	−14.52	NS
	10 nM MT3	9.884 ± 1.270	−61.22	<0.001	8.9 ± 1.771	−26.99	NS
	100 nM MT3	3.879 ± 0.791	−78.13	<0.001	5.827 ± 0.489	−52.20	=0.002
OT	Control	29.65 ± 1.640			9.134 ± 0.302		
	1 nM MT3	21.68 ± 2.434	−18.30	=0.003	8.757 ± 0.234	−4.13	NS
	10 nM MT3	6.838 ± 0.700	−67.82	<0.001	7.729 ± 1.025	−15.38	NS
	100 nM MT3	2.501 ± 0.067	−82.73	<0.001	5.306 ± 0.152	−41.91	=0.001

*Statistical significance determined using one-way ANOVA with post-hoc Sidak analysis. MT3, MT3 toxin. For other abbreviations see text*.

### Binding of ^3^H-AFDX-384 in the presence of pirenzepine and M_1_MR specific toxin MT7 in WT and M_4_ KO mice

Incubation with pirenzepine (Table 7) showed dose-dependent reduction of ^3^H-AFDX-384 binding both in WT and M_4_ KO mice. Irreversible, and very specific toxin MT7 also dose-dependently reduced ^3^H-AFDX-384 binding in both WT and M_4_ KO mice (see Table 8).

**Table 7:** Binding and percentage difference in ^3^H-AFDX 384 binding with pirenzepine in M_4_WT and M_4_KO animals vs. control (no pirenzepine).

**Brain area**		**M_4_WT (nCi/mg)**	**M_4_KO (nCi/mg)**	**WT vs. KO (%)**	***P*-value**	**M_4_WT control vs. M_4_ WT PIR**	**P-value**	**M_4_ KO control vs. M_4_ KO PIR**	***P*-value**
MOCx	Control	20.310 ± 0.389	13.96 ± 0.285	−31.27	=0.0002				
	10 nM PIR	17.89 ± 1.835	10.2 ± 0.490	−42.98	=0.0155	−11.92	NS	−26.93	<0.001
	100 nM PIR	11.56 ± 2.226	6.387 ± 0.081	−44.75	NS	−43.08	=0.01	−54.25	<0.001
SSCx	Control	23.210 ± 1.275	14.61 ± 0.487	−37.05	=0.0041				
	10 nM PIR	21.22 ± 3.075	11.21 ± 0.690	−47.17	=0.0336	−8.57	NS	−23.27	<0.001
	100 nM PIR	12.93 ± 2.132	6.525 ± 0.134	−49.54	=0.0401	−44.29	=0.022	−55.34	<0.001
VisCx	Control	22.480 ± 0.936	13.61 ± 0.503	−39.46	=0.0011				
	10 nM PIR	20.35 ± 2.271	9.741 ± 0.598	−52.13	=0.0107	−9.48	NS	−28.43	<0.001
	100 nM PIR	11.27 ± 1.824	6.166 ± 0.202	−45.29	=0.0496	−49.87	=0.003	−54.70	<0.001
Hipp	Control	16.830 ± 0.75	12.87 ± 0.366	−23.53	=0.009				
	10 nM PIR	13.57 ± 1.581	7.637 ± 0.492	−43.72	=0.0231	−19.37	NS	−40.66	<0.001
	100 nM PIR	7.402 ± 1.23	3.507 ± 0.069	−52.62	=0.0341	−56.02	<0.001	−72.75	<0.001
CA1	Control	20.640 ± 0.495	15.66 ± 0.603	−24.13	=0.0031				
	10 nM PIR	16.35 ± 1.756	9.162 ± 0.698	−43.96	=0.019	−20.78	NS	−41.49	<0.001
	100 nM PIR	8.91 ± 1.556	4.185 ± 0.016	−53.03	=0.0384	−56.83	<0.001	−73.28	<0.001
CA3	Control	15.830 ± 0.59	13.71 ± 0.433	−13.39	=0.0437				
	10 nM PIR	13.45 ± 1.65	8.743 ± 0.728	−35.00	NS	−15.03	NS	−36.23	<0.001
	100 nM PIR	8.3 ± 1.437	4.491 ± 0.035	−45.89	NS	−47.57	=0.0006	−67.24	<0.001
DG	Control	14.120 ± 0.934	10.96 ± 0.217	−22.38	=0.0303				
	10 nM PIR	10.76 ± 1.625	6.024 ± 0.504	−44.01	=0.0497	−23.80	NS	−45.04	<0.001
	100 nM PIR	5.185 ± 0.856	2.174 ± 0.089	−58.07	=0.0249	−63.28	<0.001	−80.16	<0.001
CPu	Control	33.670 ± 1.684	8.953 ± 0.301	−73.41	=0.0001				
	10 nM PIR	30.9 ± 4.385	6.062 ± 0.271	−80.38	=0.0048	−8.23	NS	−32.29	<0.001
	100 nM PIR	16.68 ± 3.283	2.733 ± 0.251	−83.62	=0.0133	−50.46	=0.011	−69.47	<0.001
NAc	Control	33.230 ± 1.947	10.25 ± 0.418	−69.15	=0.0003				
	10 nM PIR	29.9 ± 5.43	6.884 ± 0.032	−76.98	=0.0133	−10.02	NS	−32.84	<0.001
	100 nM PIR	16.24 ± 3.941	3.311 ± 0.152	−79.61	=0.0306	−51.13	=0.031	−67.70	<0.001
TH	Control	11.460 ± 0.571	7.224 ± 0.179	−36.96	=0.0021				
	10 nM PIR	10.22 ± 2.177	6.239 ± 0.468	−38.95	NS	−10.82	NS	−13.64	=0.04
	100 nM PIR	7.757 ± 1.203	3.615 ± 0.043	−53.40	=0.0263	−32.31	NS	−49.96	<0.001
MY	Control	13.670 ± 0.617	11.08 ± 1.107	−18.95	NS				
	10 nM PIR	13.58 ± 1.278	11.03 ± 1.926	−18.78	NS	−0.66	NS	−0.45	NS
	100 nM PIR	10.48 ± 2.456	5.237 ± 0.819	−50.03	NS	−23.34	NS	−52.73	<0.001
Pons	Control	15.110 ± 0.079	13.51 ± 1.279	−10.59	NS				
	10 nM PIR	14.34 ± 1.558	10.55 ± 1.058	−26.43	NS	−5.10	NS	−21.91	NS
	100 nM PIR	11.15 ± 1.414	6.253 ± 0.806	−43.92	=0.0396	−26.21	NS	−53.72	=0.02
OT	Control	28.830 ± 2.339	8.797 ± 0.335	−69.49	=0.0011				
	10 nM PIR	25.86 ± 4.201	5.554 ± 0.225	−78.52	=0.0085	−10.30	NS	−36.86	<0.001
	100 nM PIR	14.81 ± 3.031	3.647 ± 0.087	−75.37	=0.0212	−48.63	=0.029	−58.54	<0.001

*Statistical significance determined using one-way ANOVA with post-hoc Sidak analysis or Student unpaired t-test (WT. vs. KO). PIR. pirenzepine. For other abbreviations see text*.

**Table 8:** Binding and percentage difference in ^3^H-AFDX 384 binding with MT7 toxin in M_4_WT, and M_4_KO animals vs. control (no MT7 toxin).

**Brain area**		**M_4_WT (nCi/mg)**	**M_4_KO (nCi/mg)**	**WT vs. KO (%)**	***P*-value**	**M_4_WT control vs. M_4_WT MT7**	***P*-value**	**M_4_ KO control vs. M_4_KO MT7**	***P*-value**
MOCx	Control	24.600 ± 1.55	14.36 ± 1.078	−41.63	=0.0056				
	1 nM MT7	20.01 ± 0.683	13.13 ± 0.721	−34.38	=0.0023	−18.66	=0.008	−8.57	NS
	10 nM MT7	18.59 ± 1.079	10.47 ± 0.347	−43.68	=0.002	−24.43	=0.003	−27.09	=0.004
	100 nM MT7	12.72 ± 0.478	8.829 ± 0.429	−30.59	=0.0037	−48.29	<0.001	−38.52	<0.001
SSCx	Control	28.820 ± 3.316	15.46 ± 0.310	−46.36	=0.0116				
	1 nM MT7	22.66 ± 1.221	13.65 ± 0.992	−39.76	=0.0046	−21.37	=0.032	−11.71	NS
	10 nM MT7	20.55 ± 0.484	11.52 ± 0.492	−43.94	=0.0002	−28.70	=0.014	−25.49	=0.002
	100 nM MT7	13.76 ± 0.393	9.774 ± 0.660	−28.97	=0.0066	−52.26	<0.001	−36.78	<0.001
VisCx	Control	26.070 ± 2.892	14.66 ± 0.404	−43.77	=0.0175				
	1 nM MT7	19.86 ± 1.131	12.59 ± 0.943	−36.61	=0.0078	−23.82	=0.039	−14.12	=0.022
	10 nM MT7	19.77 ± 1.097	10.43 ± 0.286	−47.24	=0.0012	−24.17	=0.021	−28.85	<0.001
	100 nM MT7	13.23 ± 0.339	8.466 ± 0.326	−36.01	=0.0005	−49.25	<0.001	−42.25	<0.001
Hipp	Control	21.780 ± 1.544	13.92 ± 0.594	−36.09	=0.009				
	1 nM MT7	14.54 ± 1.974	10.24 ± 1.214	−29.57	NS	−33.24	=0.002	−26.44	=0.002
	10 nM MT7	11.77 ± 0.091	6.862 ± 0.019	−41.70	<0.0001	−45.96	<0.001	−50.70	<0.001
	100 nM MT7	7.866 ± 0.216	5.084 ± 0.171	−35.37	=0.0005	−63.88	<0.001	−63.48	<0.001
CA1	Control	25.060 ± 1.829	17.13 ± 0.417	−31.64	=0.0134				
	1 nM MT7	17.28 ± 2.268	13.04 ± 1.280	−24.54	NS	−31.05	=0.003	−23.88	=0.001
	10 nM MT7	14.11 ± 0.277	8.038 ± 0.066	−43.03	<0.0001	−43.70	<0.001	−53.08	<0.001
	100 nM MT7	9.703 ± 0.329	6.093 ± 0.264	−37.20	=0.001	−61.28	<0.001	−64.43	<0.001
CA3	Control	21.060 ± 1.332	14.75 ± 0.766	−29.96	=0.0148				
	1 nM MT7	15.02 ± 1.5	11.47 ± 0.885	−23.64	NS	−28.68	=0.001	−22.24	=0.003
	10 nM MT7	12.56 ± 0.164	8.534 ± 0.265	−32.05	=0.0002	−40.36	<0.001	−42.14	<0.001
	100 nM MT7	9.442 ± 0.15	6.995 ± 0.259	−25.92	=0.0012	−55.17	<0.001	−52.58	<0.001
DG	Control	19.310 ± 1.564	11.71 ± 0.831	−39.36	=0.0127				
	1 nM MT7	12.16 ± 2.077	7.982 ± 1.237	−34.36	NS	−37.03	=0.002	−31.84	=0.004
	10 nM MT7	8.006 ± 0.369	4.122 ± 0.126	−48.51	=0.0006	−58.54	<0.001	−64.80	<0.001
	100 nM MT7	5.174 ± 0.146	2.891 ± 0.228	−44.12	=0.0011	−73.21	<0.001	−75.31	<0.001
CPu	Control	43.410 ± 2.14	9.207 ± 0.708	−78.79	=0.0001				
	1 nM MT7	36.24 ± 2.251	7.941 ± 0.775	−78.09	=0.0003	−16.52	=0.013	−13.75	NS
	10 nM MT7	33.01 ± 0.556	5.169 ± 0.109	−84.34	<0.0001	−23.96	=0.002	−43.86	<0.001
	100 nM MT7	20.24 ± 1.477	4.422 ± 0.498	−78.15	=0.0005	−53.37	<0.001	−51.97	<0.001
NAc	Control	42.830 ± 2.79	11.28 ± 0.934	−73.66	=0.0004				
	1 nM MT7	37.61 ± 2.622	9.917 ± 1.342	−73.63	=0.0007	−12.19	NS	−12.08	NS
	10 nM MT7	28.85 ± 1.266	5.076 ± 0.165	−82.41	<0.0001	−32.64	NS	−55.00	<0.001
	100 nM MT7	18.52 ± 1.236	4.323 ± 0.410	−76.66	=0.0004	−56.76	NS	−61.68	<0.001
TH	Control	14.750 ± 1.189	7.696 ± 0.574	−47.82	=0.0059				
	1 nM MT7	12.03 ± 0.894	7.895 ± 0.271	−34.37	=0.0115	−18.44	NS	2.59	NS
	10 nM MT7	12.55 ± 0.189	7.525 ± 0.181	−40.04	<0.0001	−14.92	NS	−2.22	NS
	100 nM MT7	8.785 ± 0.235	6.125 ± 0.236	−30.28	=0.0013	−40.44	<0.001	−20.41	=0.024
MY	Control	16.190 ± 1.489	15.27 ± 1.625	−5.68	NS				
	1 nM MT7	15.98 ± 0.165	15.87 ± 1.658	−0.69	NS	−1.30	NS	3.93	NS
	10 nM MT7	15.91 ± 0.526	13.79 ± 1.579	−13.32	NS	−1.73	NS	−9.69	NS
	100 nM MT7	12.67 ± 0.393	10.61 ± 1.615	−16.26	NS	−21.74	=0.03	−30.52	NS
Pons	Control	15.030 ± 2.633	12.79 ± 2.748	−14.90	NS				
	1 nM MT7	14.3 ± 1.235	12.49 ± 2.741	−12.66	NS	−4.86	NS	−2.35	NS
	10 nM MT7	14.52 ± 1.612	11.97 ± 2.159	−17.56	NS	−3.39	NS	−6.41	NS
	100 nM MT7	11.45 ± 1.458	9.407 ± 2.037	−17.84	=0.0226	−23.82	NS	−26.45	NS
OT	Control	36.610 ± 3.097	9.624 ± 0.857	−73.71	=0.0011				
	1 nM MT7	31.46 ± 2.283	6.451 ± 0.725	−79.49	=0.0005	−14.07	NS	−32.97	=0.003
	10 nM MT7	26.16 ± 1.063	4.969 ± 0.219	−81.01	<0.0001	−28.54	=0.006	−48.37	<0.001
	100 nM MT7	16.16 ± 0.13	4.094 ± 0.323	−74.67	<0.0001	−55.86	<0.001	−57.46	<0.001

*Statistical significance determined using one-way ANOVA with post-hoc Sidak analysis or Student unpaired t-test (WT vs. KO). MT7, MT7 toxin. For other abbreviations see text*.

### Binding of ^3^H-QNB in WT and M_1_ KO, M_2_ KO, and M_4_ KO mice

#### M_1_ KO mice

**Figure 3:**
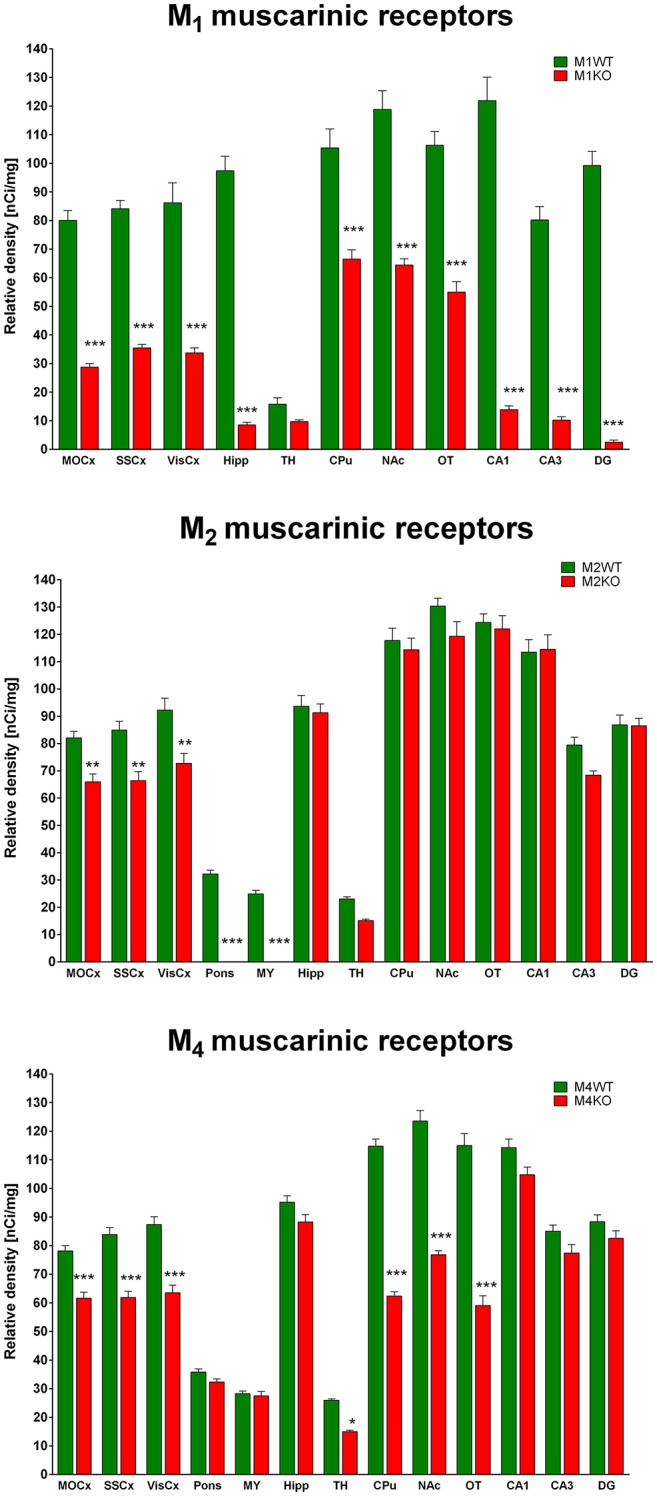
Changes in ^3^H-QNB specific binding in M_1_KO mice (**top**), M_2_KO mice (**middle**), and M_4_KO mice (**bottom**) when compared to WT animals. Non-specific binding was determined in the presence of 10 μM atropine sulfate. Ordinate: brain areas [motor cortex (MOCx), somatosensory cortex (SSCx), visual cortex (VisCx), medulla oblongate (MY), pons Varoli (pons), striatum (Caudatum-Putamen, CPu), nucleus accumbens (NAc), thalamus (TH), hippocampus (Hipp) and its specific areas CA1, CA3 and dentate gyrus (DG), olfactory tubercle (OT)], abscissa: relative density [nCi/mg]. WT, wild type; KO, knockout mice. ^*^p < 0.05, ^**^p < 0.01, ^***^p < 0.001.

Binding decrease in M_1_ KO mice (Figure 3, top) showed high densities of M_1_ MR in almost all brain areas. In cortical structures, there was approximately 60% of M_1_ MR (64, 58, and 61% in MoCx, SSCx, and VisCx, respectively). The highest density of M_1_ MR was found in hippocampus (91%) and in hippocampal regions (CA1: 89%, CA3 88%, dentate gyrus 98%, respectively). In striatum, there was 37%. Similar density of M_1_ MR was found ventral striatum, i.e., in NAc (46%) and OT (48%).

#### M_2_ KO mice

Binding decrease in M_2_ KO mice (Figure 3, middle) showed relatively low density of M_2_ MRs in MoCx, SSCx, and VisCx (20, 22, 21%, respectively). In pons and medulla oblongata, there was 100% decrease suggesting pure M_2_ MR population in these regions.

#### M_4_ KO mice

The decrease in binding in M_4_ KO mice (Figure 3, bottom) revealed 21, 26, and 27% of M_4_ MRs in MoCx, SSCx, and VisCx, respectively. The population of M_4_ MRs is represented by 43% of total number of MRs in the thalamus. However, the muscarinic population in thalamus is low. The highest proportion of M_4_ MRs was found in CPu, NAc, OT, where it represents 46, 38, and 49%, respectively.

## Discussion

Here we used knockout proof concept to ascertain the selectivity of well-established and commonly used autoradiography protocols for labeling of putative M_2_ and M_1_MR. Putative M_2_ and M_1_MR were labeled with ^3^H-AFDX-384 and ^3^H-pirenzepine, respectively. We compared the pattern and relative density of ^3^H-AFDX-384 and ^3^H-pirenzepine specific binding sites in WT with M_1_, M_2_, and M_4_ KO mice.

Our results demonstrate that ^3^H-pirenzepine labels predominantly, albeit not exclusively, M_1_MR. Thus, according to our results, ^3^H-pirenzepine can be used as M_1_MR selective ligand in brain cortex (MOCx, SSCx, VisCx) and hippocampus, in which more than 94% of ^3^H-pirenzepine binding sites are attributable to M_1_MR. In the striatum and olfactory tubercle 10–13% of ^3^H-pirenzepine specific binding sites correspond to another MR subtype. Apart from these limitations, ^3^H-pirenzepine can be considered (in concentration 5 nmol/l and in the protocol described in Methods section) as M_1_MR specific ligand.

To test whether ^3^H-pirenzepine binds also to M_4_MR, we performed also ^3^H-pirenzepine autoradiography in M_4_ KO mice, (see Table 1 for affinities). We assumed, that in case that ^3^H-pirenzepine binds M_4_MR, there should be decrease in ^3^H-pirenzepine binding in M_4_ KO mice. Indeed, ^3^H-pirenzepine binding in M_4_KO mice is decreased in cortical areas and in caudate putamen. This result can be explained, however by two ways. Firstly, ^3^H-pirenzepine binds to M_4_MR. Secondly, M_1_MR are decreased in M_4_ KO mice. M_4_MR are enriched in the striatum and expressed at modest level in cortex (Gomeza et al., 1999b). Therefore, the decrease of ^3^H-pirenzepine binding due to the lack of M_4_MR should be mostly seen in striatum and not in the cortex. We showed that there is decrease in ^3^H-pirenzepine binding by 11% in striatum of M_4_ KO mice and that the residual binding of ^3^H-pirenzepine in M_1_ KO mice is 10–13%. This suggest that indeed, in striatum ^3^H-pirenzepine binds also to M_4_MR. In contrast, there was more than 95% reduction of ^3^H-pirenzepine binding in cortex of M_1_ KO mice. The density of M_4_MR in the cortex is approximately three-fold lower than in the striatum, but decrease in ^3^H-pirenzepine binding in cortex of M_4_ KO mice was similar to that in striatum. Taken together, decrease in ^3^H-pirenzepine binding in the cortex of M_4_ KO mice suggests rather alteration in the density of M_1_ MR than binding to M_4_MR. Once again and taking above mentioned results into account, we can consider ^3^H-pirenzepine as M_1_MR highly specific ligand.

In contrast, ^3^H-AFDX-384 has poor selectivity toward M_2_MR and labels mixed population of MR in a brain area dependent manner. Autoradiography in M_2_ and M_4_KO mice showed that ^3^H-AFDX-384 binds to multiple MR populations and that this population differs between brain areas. Moreover, ^3^H-AFDX-384 binds to M_4_MR subtype in similar way as to M_2_MR subtype what is in contrast to opinion that ^3^H-AFDX-384 is M_2_MR preferential ligand. Our working hypothesis was that in case of ^3^H-AFDX-384 high selectivity toward M_2_MR, gene deletion of M_2_MR resulting in absence of M_2_MR protein in M_2_KO mice should result in complete loss of ^3^H-AFDX-384-specific binding. Even though ^3^H-AFDX-384 specific binding was reduced in M_2_KO mice throughout the brain, the reduction was far less than expected. Surprisingly ^3^H-AFDX-384 specific binding was also reduced in M_4_KO mice. In brain regions rich in M_4_MR, such as striatum the ^3^H-AFDX-384 specific binding was reduced more than 70%. This suggest that^3^H-AFDX-384 binds to M_4_MR subtype as well. Even though affinity of AFDX-384 is high (see Table 1), the extent of ^3^H-AFDX-384 binding to M_4_MR in vitro autoradiography is surprising. In order to increase the selectivity of ^3^H-AFDX-384 toward M_2_MR, we tried to block ^3^H-AFDX-384 binding to M_4_MR by addition of antagonist PD102807 (with pKi higher by two orders of magnitude for M_4_MR than for other MR subtypes: 7.3–7.4 vs. 5.2–6.7, see Table 1). Assuming the high selectivity of PD102807 for M_4_MR and binding of ^3^H-AFDX-384 also to M_4_MR, PD102807 should markedly reduce the residual ^3^H-AFDX-384 binding in M_2_KO mice. Indeed, addition of PD102807 into incubation medium dose-dependently reduced the residual ^3^H-AFDX-384 specific binding in M_2_KO mice, suggesting effective blocking of putative M_4_MR. At the highest concentration (1,000 nmol/l), there was no residual binding of ^3^H-AFDX-384 in M_2_KO mice. To test the potential binding capacity of PD102807 to other MR subtypes we performed similar experiment in M_4_KO mice. In case that PD102807 is selective M_4_MR ligand, adding PD102807 to the incubation medium should not interfere with ^3^H-AFDX-384 with binding capacity in M_4_KO mice, since there are no M_4_MR. However, PD102807 also dose-dependently reduced ^3^H-AFDX-384 specific binding in M_4_KO mice with no M_4_MR, indicating that under our experimental conditions PD102807 has poor selectivity toward M_4_MR. Assuming that ^3^H-AFDX-384 predominantly labels M_2_MR and M_4_MR and no other MR subtype our results suggest, that PD102807 binds to both M_4_MR and M_2_MR. We can therefore conclude that co-incubation with PD102807 is not a suitable protocol for M_4_MR binding elimination and M_2_MR specific binding autoradiography. Thus, we explored another specific binding antagonist—MT3 toxin from Dendroaspis angusticeps venom—with effort to eliminate binding to M_4_MR. This toxin is believed to block binding to M_4_MR with great order of magnitude difference to other MR subtypes (pKi = 8.7 (M_4_MR) vs. < 6 in M_2_, M_3_, M_5_MR and 7.1 in M1MR, see Table 1). Similarly to PD102807, MT3 toxin was unable to effectively block ^3^H-AFDX-384 binding to M_4_MR. There remained ^3^H-AFDX-384 binding capacity in M_2_ KO mice in the highest (100 nmol/l) MT3 toxin concentration which should be able to block all M_4_MR. Moreover, similarly to PD102807, MT3 not only dose-dependently decreased residual binding of ^3^H-AFDX-384 in M_2_ KO mice, but also in M_4_ KO mice. This suggest that MT3 toxin does not distinguish between M_4_ and M_2_MR, at least under our experimental conditions. Finally, we tested the hypothesis that ^3^H-AFDX-384 binds also to M_1_MR. We tested our hypotheses in M_4_ KO animals using two approaches: binding in the presence of pirenzepine (see Table 1 for affinities) and irreversible, and very specific toxin MT7 (with pKi = 9.8 to M_1_MR and pKi <6 to other muscarinic subtypes). We choose M_4_ KO mice in order to exclude possible binding of pirenzepine toward M_4_MR in striatum as discussed above. Even at 10 nM concentration, pirenzepine significantly reduced ^3^H-AFDX-384 specific binding in M_4_ KO mice, suggesting that ^3^H-AFDX-384 binds to a certain extent also M_1_MR. Moreover, irreversible, and very specific toxin MT7 showed dose-dependent reduction of ^3^H-AFDX-384 specific binding in M_4_ KO mice further supporting binding of ^3^H-AFDX-384 to M_1_MR. It is therefore possible to conclude that ^3^H-AFDX-384 is not suitable ligand for M_2_MR. Moreover, we were not successful in designing any protocol to block M_4_ or M_1_MR and increase the selectivity of ^3^H-AFDX-384 autoradiography of M_2_MR.

All five MR subtypes are highly expressed in the brain (Levey et al., 1991; Wess et al., 2003; Oki et al., 2005). The M_1_ and M_4_ MRs represent the most abundant subtypes with the highest expression in the cortex, hippocampus and striatum that can be well illustrated using ^3^H-QNB autoradiography in our M_1_ KO and M_4_ KO mice.

*In vitro* receptor autoradiography has been used for decades for mapping anatomical distribution and quantification of broad range of receptors (Manuel et al., 2015; Farar and Myslivecek, 2016). The standard autoradiography is based on equilibrium binding of radioactively labeled antagonist. The key aspect of autoradiography is thus the selectivity of radioligand toward its target. MR subtypes show often overlapping pattern of expression and most MR ligands have poor selectivity, making the discrimination of individual subtypes difficult. While there are several ligands available, which specifically label MR, they do not distinguish individual MR subtypes. *In vitro* heterologous expression systems have helped to describe the affinity of a broad range of antagonists toward each of the five MR (Buckley et al., 1989; Dörje et al., 1991; Dong et al., 1995). These studies have identified so-called preferential MR ligands such as AFDX-384 and pirenzepine, which are commonly used to target putative M_2_ and M_1_MR, respectively. AFDX-384 has the highest affinity toward M_2_MR, but also similar affinity toward M_4_MR (Dörje et al., 1991).

We have proofed here that autoradiography protocol for ^3^H-pirenzepine is suitable for M_1_MR detection (with limitations in CPu, NAc and OT) where pirenzepine binds also by approximately 10% to another MR subtype, likely M_4_MR). Thus, this ligand can be used as M_1_MR specific as before (e.g., Wamsley et al., 1984; McCabe et al., 1987; Farar and Myslivecek, 2016).

On the other hand, ^3^H-AFDX-384 is not suitable to detect M_2_MR as believed previously (Entzeroth and Mayer, 1990; Wolff et al., 2008; Grailhe et al., 2009). However, there are some studies that previously correctly defined ^3^H-AFDX-384 as M_2_ partially selective (Mulugeta et al., 2003) or as M_2_/M_4_ ligand (Nieves-Martinez et al., 2012).

The fact that ^3^H-AFDX-384 is not selective toward M_2_MR was predictable in the light of AFDX-384 affinity to MR subtypes (see Table 1). However, almost identical binding to M_2_MR and M_4_MR is a new finding. We also tried to find specific protocol for M_2_MR specific binding using different antagonists (PD102807, MT3 toxin, pirenzepine, and MT7 toxin). None of these antagonists were able to completely block other receptors. And thus it is necessary to conclude that there is no way to make the binding more specific to M_2_MR.

Another aspect of our data is demonstration of M_1_MR, M_2_MR, and M_4_MR distribution. It can be deduced from Figure 1 that M_1_MR are not as hugely present in the cortical structures as in some hippocampal areas (dorsal hippocampus, CA1 area, and dentate gyrus). Very low M_1_MR density is in the thalamus. Comparing binding in M_2_WT and M_2_KO mice we can conclude that there is relatively high density of M_2_MR in cortical structures, medulla oblongata, pons and thalamus. In contrast, hippocampus and striatum does not have high amount of M_2_MR. Striatum is, however, rich in M_4_MR similarly to nucleus accumbens and olfactory tubercles. Also, the density of M_4_MR in the cortex is relatively high. However, one should take into account that these data represent relative proportions of respective MR subtypes, since the binding was determined in radioligand concentrations around K_D_ and the receptors were not saturated. ^3^H-pirenzepine has pK_D_ around 7.9 (Watson et al., 1984), ^3^H-AFDX 384 has KD between 3 and 4 nmol.l^−1^ (Castoldi et al., 1991) what is comparable to ^3^H-pirenzepine. In order to verify the proportion of respective MR subtypes we have used specific MR knockouts (M_1_, M_2_, and M_4_ KOs) and measured a decrease in non-specific radioligand (^3^H-QNB) binding. This radioligand has much higher affinity to MR and pK_D_ ranging between 10.6 and 10.8 (Peralta et al., 1987). These experiments showed the highest proportion (usually above 50%) of M_1_MR in virtualy all studied brain areas. M_2_MR take up to 20% in cortical areas and 34% in thalamus. M_4_MR were abundant (40% approximately) in thalamus, striatum and ventral striatum (NAc and OT), about 20% of M_4_MR can be found in cortical structures. This is in general agreement with previously published data (Levey et al., 1991; Wess et al., 2003; Oki et al., 2005), although we have obtained slightly different pattern of MR subtypes distribution what can be caused by the use of different radioligand. Some discrepancies between results could be attributed to the fact that not all antibodies are selective (Pradidarcheep and Michel, 2016). As referenced by Manuel et al. (2015) relatively good correlation exists between the radioligand detected receptor number and the immunolabeled receptor protein for M_1_ subtype.

Our results concerning the MR distribution are generally in in agreement with previous study (Oki et al., 2005) which employed different muscarinic knockouts but used non-specific radioligand (^3^H-NMS). However, in this study less brain areas were investigated and different method (direct radioligand binding) used. We sldo found similar pattern of MR subtype distribution as investigated using antibodies and electron microscopy (Hersch et al., 1994), immunoprecipitation (Levey et al., 1991; Tice et al., 1996) and radioligand binding (Flynn and Mash, 1993). Another study (Ferrari-Dileo et al., 1994) that used selective labeling found similar pattern of M_4_MR distribution as here. All five MR subtypes are highly expressed in the brain (Levey, 1996; Oki et al., 2005). The M_1_ and M_4_ MR represent the most abundant subtypes with the highest expression in the cortex, hippocampus and striatum that can be well illustrated using ^3^H-QNB autoradiography in our M_1_ KO and M_4_ KO mice. Previous research has shown that MRs, mostly M_1_ and M_4_ MRs, might be an interesting pharmacological target for the treatment of neurodegenerative and neuropsychiatric diseases such as Alzheimer's disease, Parkinson's disease, schizophrenia, depression and also drug abuse (Bodick et al., 1997; Scarr et al., 1999; Brady et al., 2008; Langmead et al., 2008; Bradley et al., 2017; Dall et al., 2017). Therefore, *in vitro* radioligand binding studies, direct and indirect, represent an important pharmacological tool to precisely characterize the involvement of specific MR subtypes in such a disease as well as to study the binding properties, affinity and efficacy of new chemical compounds with the potential to become a selective drug toward the receptor.

## Conclusion

We can therefore conclude that ^3^H-pirenzepine showed high selectivity toward M_1_MR and can be used with minor limitations as M_1_MR specific ligand. In contrast, ^3^H-AFDX-384 binding sites represent different populations of MR subtypes which is brain-region-specific. This finding has to be taken into account when interpreting the binding data. Our experiments with ^3^H-QNB binding decrease in M_1_, ${\text{M}}_{2^{\cdot \cdot }}$, and M_4_ KO animals showed the highest proportion of M_1_MR in virtualy all studied brain areas. M_2_MR were expressed in cortical areas and in thalamus. M_4_MR were abundant in thalamus, striatum and ventral striatum (NAc and OT as well as in cortical structures.

## Author contributions

JM and VF contributed to the conception and design of the reported studies. PV, IK, and VF conducted all of the experiments. SF, VF, and PV analyzed the data. PV and VF contributed to the drafting and revision of the manuscript. JM wrote the manuscript in the final form. All authors approved the final version and agreed to be accountable for all aspects of the work.

### Conflict of interest statement

The authors declare that the research was conducted in the absence of any commercial or financial relationships that could be construed as a potential conflict of interest. The reviewer AB and handling Editor declared their shared affiliation.
